# Covert Consciousness in Acute Brain Injury Revealed by Automated Pupillometry and Cognitive Paradigms

**DOI:** 10.1007/s12028-024-01983-7

**Published:** 2024-04-11

**Authors:** Marwan H. Othman, Markus Harboe Olsen, Karen Irgens Tanderup Hansen, Moshgan Amiri, Helene Ravnholt Jensen, Benjamin Nyholm, Kirsten Møller, Jesper Kjaergaard, Daniel Kondziella

**Affiliations:** 1 Department of Neurology, Rigshospitalet, Copenhagen University Hospital, Inge Lehmanns Vej 8, 2100 Copenhagen, Denmark; 2 Department of Neuroanesthesiology, Rigshospitalet, Copenhagen University Hospital, Copenhagen, Denmark; 3 Faculty of Health Science, University of Southern Denmark, Odense, Denmark; 4 Department of Cardiology, Rigshospitalet, Copenhagen University Hospital, Copenhagen, Denmark; 5 Department of Clinical Medicine, University of Copenhagen, Copenhagen, Denmark

**Keywords:** Cardiac arrest, Cognitive motor dissociation, Coma, Intensive care medicine, Traumatic brain injury

## Abstract

**Background:**

Identifying covert consciousness in intensive care unit (ICU) patients with coma and other disorders of consciousness (DoC) is crucial for treatment decisions, but sensitive low-cost bedside markers are missing. We investigated whether automated pupillometry combined with passive and active cognitive paradigms can detect residual consciousness in ICU patients with DoC.

**Methods:**

We prospectively enrolled clinically low-response or unresponsive patients with traumatic or nontraumatic DoC from ICUs of a tertiary referral center. Age-matched and sex-matched healthy volunteers served as controls. Patients were categorized into clinically unresponsive (coma or unresponsive wakefulness syndrome) or clinically low-responsive (minimally conscious state or better). Using automated pupillometry, we recorded pupillary dilation to passive (visual and auditory stimuli) and active (mental arithmetic) cognitive paradigms, with task-specific success criteria (e.g., ≥ 3 of 5 pupillary dilations on five consecutive mental arithmetic tasks).

**Results:**

We obtained 699 pupillometry recordings at 178 time points from 91 ICU patients with brain injury (mean age 60 ± 13.8 years, 31% women, and 49.5% nontraumatic brain injuries). Recordings were also obtained from 26 matched controls (59 ± 14.8 years, 38% women). Passive paradigms yielded limited distinctions between patients and controls. However, active paradigms enabled discrimination between different states of consciousness. With mental arithmetic of moderate complexity, ≥ 3 pupillary dilations were seen in 17.8% of clinically unresponsive patients and 50.0% of clinically low-responsive patients (odds ratio 4.56, 95% confidence interval 2.09–10.10; *p* < 0.001). In comparison, 76.9% healthy controls responded with ≥ 3 pupillary dilations (*p* = 0.028). Results remained consistent across sensitivity analyses using different thresholds for success. Spearman’s rank analysis underscored the robust association between pupillary dilations during mental arithmetic and consciousness levels (rho = 1, *p* = 0.017). Notably, one behaviorally unresponsive patient demonstrated persistent command-following behavior 2 weeks before overt signs of awareness, suggesting prolonged cognitive motor dissociation.

**Conclusions:**

Automated pupillometry combined with mental arithmetic can identify cognitive efforts, and hence covert consciousness, in ICU patients with acute DoC.

**Supplementary Information:**

The online version contains supplementary material available at 10.1007/s12028-024-01983-7.

## Introduction

Each year, an estimated 2 in 1,000 people fall into a coma and enter the intensive care unit (ICU) [1]. Of all patients who do not immediately recover and show overt signs of consciousness, around 15–20% may be awake and aware despite being clinically unresponsive [2, 3]; this condition is known as cognitive motor dissociation (CMD) [4, 5].

Identifying CMD and other forms of residual consciousness is extremely challenging in the ICU [3, 6–8], but it is of paramount importance for several reasons: first, residual consciousness is overlooked in 40% of patients with disorders of consciousness (DoC) [9], potentially putting these patients at risk because life-sustaining treatment is often withdrawn when the prognosis is perceived to be poor [10, 11]. Second, the concept of residual consciousness creates new opportunities because patients with CMD have better outcomes at 1 year post injury than genuinely unresponsive patients [5, 8]. Thus, identifying residual consciousness after acute brain injury is crucial for prognostication, treatment decisions, and resource allocation, but low-cost sensitive bedside markers are missing.

Interestingly, automated pupillometry can detect cognitive load by demonstrating pupillary dilations in study participants exposed to arousal or mental activity [12, 13]. For example, in awake volunteers, pupillary dilation occurs each time they engage in mental arithmetic, followed by pupillary constriction when told to relax. If this cycle is successfully repeated, say, five times [12], an individual has demonstrated command-following abilities without the need for verbal or skeletal muscle motor output. We therefore hypothesized that automated pupillometry can identify residual consciousness, and potentially CMD [5], in a subset of ICU patients with acute brain injury and DoC.

## Methods

### Study Participants

We prospectively enrolled unresponsive or low-response patients with acute traumatic and nontraumatic brain injuries admitted to the neurocritical and cardiological ICUs of a tertiary referral center. The ICUs were screened daily for patients admitted overnight, and patients were consecutively enrolled when deemed feasible by the attending clinical team and patient family consent was obtained. Using automated pupillometry, we investigated patients at least once or, if possible, several times during their ICU stay until they recovered consciousness, were discharged from the ICU, or died, whichever came first. We did tests on one eye (not both). Eye selection was random or based on practical considerations at the bedside. Participants with eye disease of any kind were excluded, as well as acute medically unstable patients. Age-matched and sex-matched healthy volunteers were recruited in parallel using a local advertisement. Each healthy volunteer was investigated once.

### Clinical Classification of Consciousness Levels

Applying previously described criteria [7], patients with DoC were classified into coma, unresponsive wakefulness syndrome (UWS), minimally conscious states (MCS) minus/plus, and emerged from MCS at each pupillary measurement. Intravenous sedation was stopped or decreased as much as possible [7], Briefly, patients were classified according to consciousness levels by a detailed neurological bedside examination performed under supervision of an experienced board-certified neurologist (DK) just prior to pupillometry. The neurological examination included (1) cranial nerves and sensorimotor status, (2) Glasgow Coma Scale, (3) Full Outline of Unresponsiveness score [14], (4) fixation and visual pursuit using a mirror, (5) ability to follow simple motor commands (including with instructions from the family, if present, to stimulate arousal), (6) reaction to central and peripheral noxious stimuli in the absence of command following, and (7) evaluation of verbal and nonverbal communication. Patients were divided into those without (≤ UWS, i.e., coma or UWS) and those with (≥ MCS, i.e., MCS or better) clinical signs of residual consciousness.

### Evaluation of Pupillary Responses to Cognitive Loads

Using a PLR-3000 pupillometer (NeurOptics, Laguna Hills, CA; sampling rate 30 Hz, accuracy ± 0.03 mm), we measured pupillary responses over 10 min (detailed video in the Supplementary Material). When measuring the responses, the examiner kept one of the patient’s eyes open. To prevent dry eyes and eye discomfort, regular rest periods with eyes closed were taken between measurements.

### Passive Cognitive Paradigms

The first stimulus was the participants’ own facial reflection in a mirror; the second a series of three different 10 s long sound clips, each with 20 s of white noise interspersed. The first sound clip was “Aaron Copland’s Rodeo—Four Dance Episodes” [15], the second sound clip was that of a crying toddler, and the third sound clip was a burglary alarm, all included for their arousal-inducing properties. We used different thresholds to classify pupil dilation as successful. For the mirror stimulus, a single pupillary dilation was sufficient. For the series of prerecorded sounds, two or more of three pupillary dilations were required for success. We compared the pupillary size during the 10 s of the stimulus to the 5 s immediately before it and to 5 s during the midresting period (so that the pupil could return to baseline after a stimulus).

### Active Cognitive Paradigms

These included two series of mental arithmetic tasks of increasing complexity. Mental arithmetic is a robust method to induce cortical activation that has been validated by studies based on functional neuroimaging and electroencephalography (EEG) [16–19]. Furthermore, we previously showed that such tasks can induce sufficient cognitive load in healthy volunteers and neurological patients detectable by automated pupillometry [12]. It is important to note that successful command following is determined by the attempt to perform a mental calculation. Thus, it is the mental effort and cognitive load that induces pupillary dilation, regardless of whether the result of the calculation is mathematically correct or not. The mental arithmetic tasks consisted of five sets of moderate and five of sets high complexity tasks (4 × 36, 8 × 32, 3 × 67, 6 × 37, and 7 × 43; and 21 × 22, 33 × 32, 55 × 54, 43 × 44, and 81 × 82, respectively). Participants were asked to engage in mental arithmetic and relax, five times in succession. Task duration was set to 20 s with 20 s rest in between, as previously described [12]. Minimal cutoffs for successful command following were defined as three or more pupillary dilations on five mental arithmetic tasks in one set (moderate or hard mental arithmetic). To test more conservative thresholds, we also included analyses using cutoffs of three or more pupillary dilations in both sets and cutoffs of four or more pupillary dilations in one and in both sets.

### Pupillary Dilation

The PLR-3000 pupillometer records pupil diameter over time and initially displays the information as a graph on the device. To evaluate pupillary dilation, we uploaded the data as an Excel file via the device’s Bluetooth functionality. We then analyzed pupillary dilations using an in-house developed R package (see the following section). A pupillary dilation was defined as a significantly larger pupil compared with the resting period before and after a stimulus/task (i.e., we calculated the average pupillary size during a stimulus and used an unpaired Student’s *t*-test to compare it against the averages from preceding and subsequent rest periods). Trigger markers of the applied stimuli were manually inserted while recording. All pupil measurements were included for each period of interest and, as stated, compared using Student’s *t*-test. Although these were autocorrelated measurements, we used the unpaired *t*-test because it is more conservative and does not require equal numbers of measurements in the groups. All recordings were visually inspected for artifacts; however, the presence or absence of pupillary dilations were revealed to the investigators only after the publication of the statistical analysis plan [20]. Data points with abrupt deviations exceeding 1.5 mm from the preceding data point (i.e., blinks) were labeled as physiologically implausible and removed. For transparency, we have added a supplemental file revealing all corrected trigger markers (Supplemental Material).

### Sample Size and Power Calculations

The study sample size was calculated based on data obtained from a feasibility study [12]. To detect a clinically significant difference in pupillary dilations between resting and stimulation periods, we aimed for a statistical power of 80% and a type 1 error probability (α) of 0.05. This calculation resulted in a minimum required sample size of *n* = 41 patients. Because the pupillometry paradigm was easy to implement in daily clinical routine and associated with neither harm nor risks, and because we also wanted to test for more conservative success thresholds, we aimed for a sample size of approximately twice that number.

### Statistics Analysis

Following a preregistered statistical analysis plan [20], statistics were performed in R (R Core Team, Vienna, Austria). Most of the patients underwent repeated assessments, with each assessment treated as an independent observation, including evaluations of the state of consciousness and pupillometry. The number of pupillary dilations of each group were compared using the *χ*^2^ test or Fisher’s exact test as appropriate. Numeric data were analyzed for intergroup comparisons using Student’s *t*-test, the Mann–Whitney *U*-test, or the Kruskal–Wallis test, as appropriate. Spearman’s rank analysis was done to assess the association between average pupillary dilations (in millimeters) during mental arithmetic and consciousness levels. A *p* ≤ 0.05 was considered significant.

### Data Sharing Statement and Code Availability

All study data are available in the article and Supplementary Material (including raw data). The algorithm for pupillometry data processing is available at https://github.com/lilleoel/clintools.

### Additional Material

Details are provided (1) in a preregistered statistical analysis plan at Zenodo.org [20]; (2) in the online Supplementary Material, including pupillometry raw data, anonymized patient data, and a videoclip of the examination setting; and (3) at https://cran.r-project.org/web/packages/clintools/, which also includes the code for processing the pupillometry data. We also provide a step-by-step guide to use automated pupillometry for the bedside detection of covert consciousness (Supplementary Methods).

### Ethics

Regional Ethics Committee (Region Hovedstaden) approval was obtained (H-21022096).

## Results

We obtained 699 pupillometry recordings at 178 time points from 91 ICU patients with brain injury (mean age 60 ± 13.8 years; 31% women; 49.5% nontraumatic brain injuries); 95.5% were ≤ UWS at first evaluation. Fifty-four participants were investigated twice, and 28 participants were investigated three times. Recordings were also obtained from 26 age-matched and sex-matched controls (59 ± 14.8 years, 38% women). Table 1 and Tables S1–S4 provide baseline statistics, individual patient data, and detailed pupillometry results.

**Table 1:** Baseline characteristics of study population

Characteristics	Controls (*n* = 26)	Patients (*n* = 91)	*p* value	≥ MCS (*n* = 37)^a^	≤ UWS (*n* = 87)^a^	OR (95% CI)	*p* value
Demographics							
Age, mean (SD) (yr)	59.0 (14.8)	60.1 (13.9)	0.872	61.2 (14.9)	60.02 (13.3)		0.690
Male, *n* (%)	16 (61.5)	63 (69.2)	0.483	27 (73.0)	59 (67.8)	1.28 (0.51–3.39)	0.672
mRS baseline, *n* (%)							
0–2	26	81 (89.0)		29 (78.4)	67 (77.0)	0.43 (0.03–6.30)	0.586
> 2	0	6 (6.6)		2 (5.4)	2 (2.3)		
Undisclosed	0	4 (4.4)		6 (16.2)	18 (20.7)		
Cause of ICU admission, *n* (%)							
Cardiac arrest	n/a	45 (49.5)		24 (64.9)	43 (49.4)	2.01 (0.86–4.87)	0.117
Traumatic brain injury	n/a	2 (2.2)		1 (2.7)	2 (2.3)	1.18 (0.02–23.32)	>0.99
Subarachnoid hemorrhage	n/a	25 (27.5)		5 (13.5)	24 (27.6)	0.41 (0.11–1.25)	0.108
Intracerebral hemorrhage	n/a	15 (16.5)		7 (18.9)	14 (16.1)	1.21 (0.38–3.61)	0.794
Subdural hemorrhage	n/a	4 (4.4)		0	4 (4.6)	0 (0–3.56)	0.316
Prior comorbidity, any, *n* (%)							
Cardiopulmonary	n/a	43 (47.3)		18 (48.6)	40 (46.0)	0.87 (0.37–2.07)	0.842
Neurological	n/a	7 (7.7)		6 (16.2)	6 (6.9)	2.59 (0.64–10.51)	0.180
Cerebrovascular	n/a	5 (5.49)		5 (13.5)	4 (4.6)	3.21 (0.65–17.24)	0.125
Epilepsy	n/a	1 (1.1)		1 (2.7)	1 (1.1)	2.37 (0.03–189.36)	0.509
Migraine	n/a	1 (1.1)		0	1 (1.1)	0 (0–91.57)	>0.99
Diabetes	n/a	7 (7.7)		1 (2.7)	6 (6.9)	0.38 (0.01–3.29)	0.673
Other medical or surgical comorbidities^b^	n/a	47 (51.6)		3 (8.1)	44 (50.6)	0.09 (0.02–0.31)	< 0.001
Clinical examinations and pupillometry sessions							
Number of clinical examinations^c^	26	178		48	130		
GCS, at clinical examination, median (min, max)	15	5 (3, 15)	< 0.001	11 (5, 15)	3 (3, 9)		< 0.001
FOUR, at clinical examination, median (min, max)	16	5 (0, 16)	< 0.001	13 (5, 16)	4 (0, 10)		< 0.001
Number of automated pupillometry examinations							
Visual	26	177		48	129		
Auditory	26	177		48	129		
Mental arithmetic (moderate)	26	177		48	129		
Mental arithmetic (hard)	26	168		41	127		
Level of sedation, during clinical examination, *n* (%)^d^							
None or minimal	n/a	75 (42.1)	< 0.001	41 (85.4)	34 (26.2)	16.21 (6.44–47.06)	< 0.001
Low to moderate	n/a	17 (9.6)	0.137	4 (8.3)	13 (10.0)	0.82 (0.18–2.84)	>0.99
High or very high	n/a	86 (48.3)	< 0.001	3 (6.3)	83 (63.8)	0.04 (0.01–0.13)	< 0.001

CI, confidence interval, FOUR, Full Outline of Unresponsiveness, GCS, Glasgow Coma Scale, ICU, intensive care unit, max, maximum, MCS, minimally conscious state, min, minimum, mRS, modified Rankin Scale, n/a, not applicable, OR, odds ratio, SD, standard deviation, UWS, unresponsive wakefulness syndrome

^a^The number includes patients in either ≤ UWS or ≥ MCS. Note that consciousness levels could vary over time and at different examinations and were therefore interpreted here as independent data

^b^Some patients had multiple comorbidities, including hypertension (*n* = 30), cardiac arrythmia (*n* = 9), ischemic heart disease (*n* = 7), renal impairment (*n* = 7), chronic obstructive pulmonary disease (*n* = 6), stroke (*n* = 5), breast cancer (*n* = 2), prostate cancer (*n* = 2), asthma (*n* = 1), and hematological malignancy (*n* = 1)

^c^Clinical examinations included, but were not limited to, GCS and FOUR: see the Methods section for details

^d^Sedation levels were stratified as previously described [17]: none or minimal (absence of fentanyl, remifentanil, propofol, midazolam, sodium thiopental, or sevoflurane), low to moderate (fentanyl levels < 500 ug/h or < 200 ug/h combined with propofol, and remifentanil levels < 1,000 ug/h or < 250 ug/h combined with propofol; or propofol < 100 mg/h, midazolam levels < 10 mg/h, and sevoflurane levels < 3%), and high or very high (propofol levels ≥ 100 mg/h, fentanyl levels ≥ 500 ug/h or ≥ 200 ug/h combined with propofol, remifentanil levels ≥ 1,000 ug/h or ≥ 250 ug/h combined with propofol, midazolam levels ≥ 10 mg/h, sevoflurane levels ≥ 3%, or any dosage of sodium thiopental [22])

### Passive Cognitive Paradigms

**Fig. 1:**
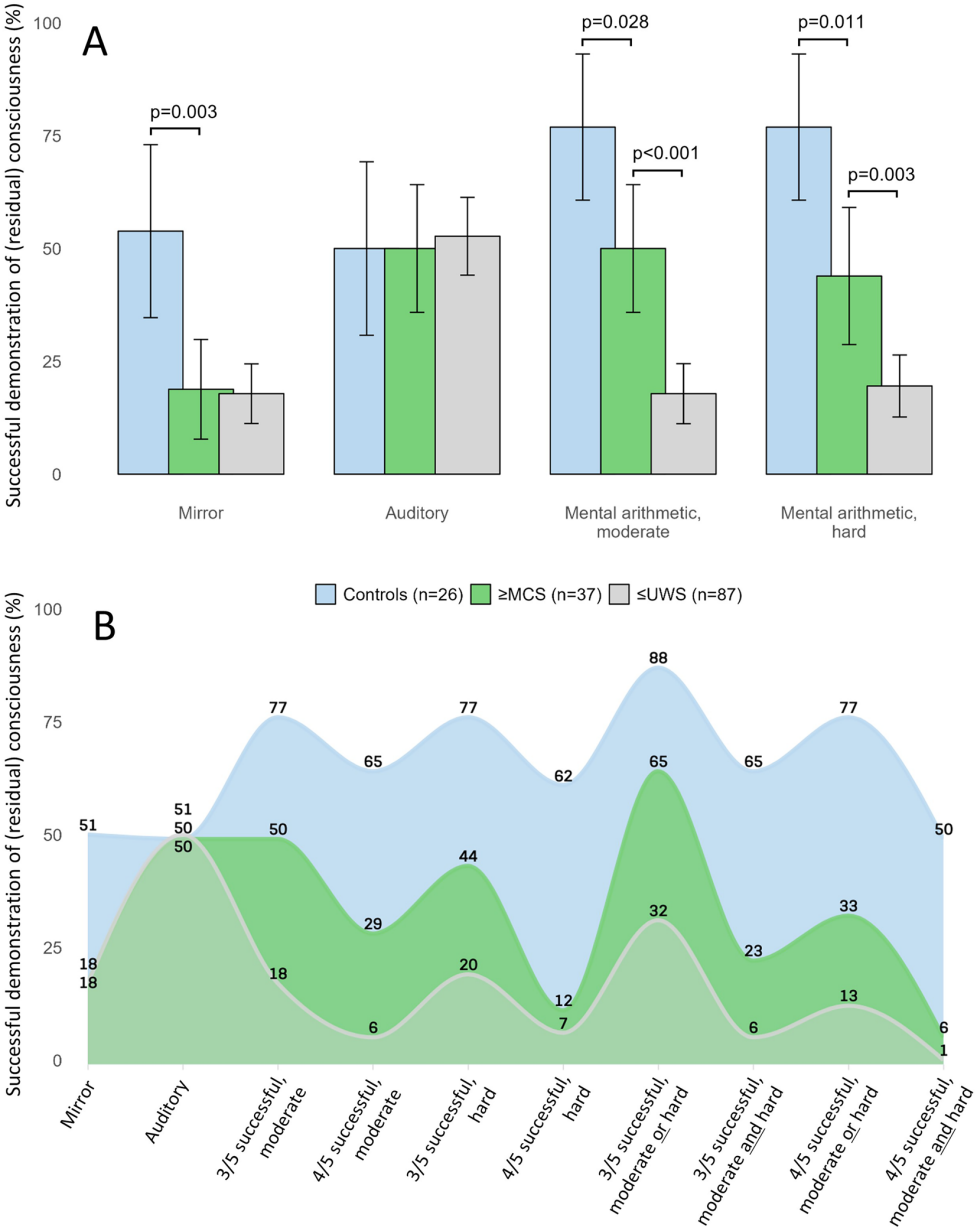
Pupillary responses to passive and active paradigms in controls, ≥ MCS patients, and ≤ UWS patients. **a** The bar chart illustrates the pupillary responses to different automated pupillometry paradigms, both passive (visual, auditory) and active (mental arithmetic), along with the corresponding number of patients and controls (%) who achieved success in each paradigm. **b** Area chart of pupillary responses (%) to passive paradigms and active task-based paradigms with adjusted thresholds for success. The area chart depicts the proportions (%) of patients with acute brain injury and controls achieving detectable pupillary dilations in response to passive visual and auditory paradigms, and active mental arithmetic paradigms. The latter included five sets of moderate and five of high complexity mental arithmetic. eMCS, emerged from minimally conscious state, MCS, minimally conscious state, UWS, unresponsive wakefulness syndrome

Pupillary dilations in the mirror paradigms differed between healthy controls and patients (Fig. 1). Controls responded to their reflection more often than ≥ MCS patients (odds ratio 4.93; 95% confidence interval 1.55–16.69, *p* = 0.003). However, there was no difference between ≤ UWS and ≥ MCS patients. Auditory stimuli were not different between healthy controls and patients, nor between ≤ UWS and ≥ MCS groups.

### Active Cognitive Paradigms

**Fig. 2:**
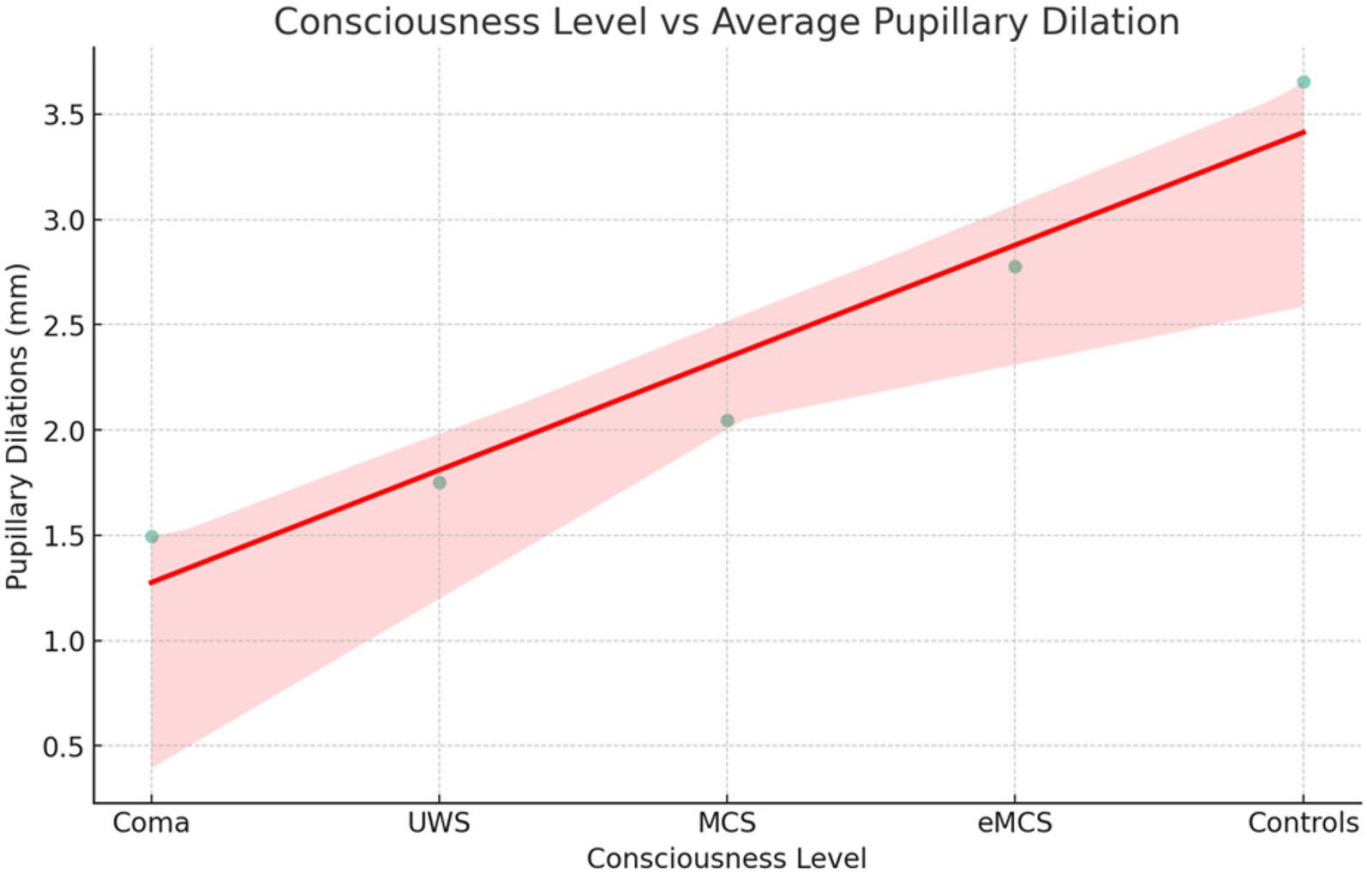
Scatter plot of the correlation between consciousness levels and average pupillary dilations during mental arithmetic. This graph visualizes the relationship between pupillary dilations during mental arithmetic in millimeters (calculated as the mean of medium-task and hard-task pupillary dilations) and clinical consciousness levels in patients and healthy controls. The red line represents a linear regression fit, indicating the trend of correlation. eMCS, emerged from minimally conscious state*,* MCS, minimally conscious state*,* UWS, unresponsive wakefulness syndrome (Color figure online)

**Fig. 3:**
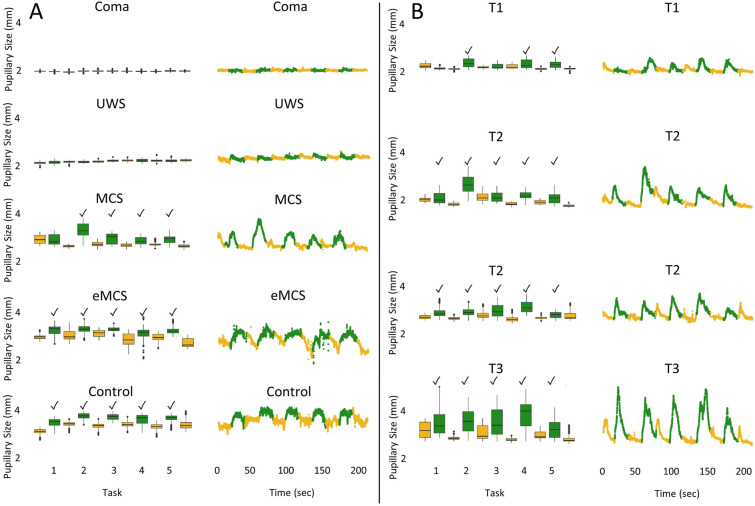
Pupillometry data from four exemplary patients in different states of consciousness and one control (**a**), as well as one patient with covert consciousness (**b**). This figure illustrates pupillometry data obtained from participants in various states of consciousness. The data reflect the count of successful pupillary dilations recorded through automated pupillometry during mental arithmetic paradigms. The figure presents data for each study participant in two distinct formats: boxplots (left) and scatter plots (right). The color code distinguishes between periods of mental arithmetic (green) and rest periods (yellow), with the *x*-axis representing time in seconds (“0–50–100–150–200”). The data indicate that pupillary sizes during mental arithmetic were significantly larger than during rest periods, indicating consistent pupillary dilation. This was observed in at least four out of the five tasks for participants in MCS, eMCS, and the healthy volunteer, as well as a patient in presumed cognitive motor dissociation (whose case description is given in Fig. 4). However, there were no significant differences between task and rest periods for the patients with coma and patients with UWS. The checkmark denotes significant pupillary dilation (*p* value < 0.0001). eMCS, emerged from minimally conscious state*,* MCS, minimally conscious state*,* UWS, unresponsive wakefulness syndrome (Color figure online)

Pupillometry with mental arithmetic could distinguish between patient groups (Fig. 1). With mental arithmetic of moderate complexity, ≥ 3 pupillary dilations were seen in 17.8% ≤ UWS patients and 50.0% ≥ MCS patients (odds ratio 4.56; 95% confidence interval 2.09–10.10, *p* < 0.001). In comparison, 76.9% healthy volunteers responded with ≥ 3 pupillary dilations during mental arithmetic, different from patients (*p* = 0.028). With increasingly complex mental arithmetic, the ability of pupillometry to distinguish between ≤ UWS patients, ≥ MCS patients, and healthy controls remained unchanged. Spearman’s rank analysis underscored the robust association between pupillary dilations during mental arithmetic and consciousness levels (rho = 1, *p* = 0.017; Fig. 2). These differences remained consistent with a more conservative cutoff point of ≥ 4 pupillary dilations per five mental arithmetic tasks in *either* one of the hard or the moderate complex set of arithmetic tasks, and in *both* hard and moderate complex set of tasks. However, with a cutoff point of ≥ 4 pupillary dilations, the percentage of success in ≤ UWS patients decreased to 6.2% and 7.1% for moderate-level and hard-level arithmetic tasks, respectively. The strictest criteria for success required ≥ 4 pupillary dilations in both sets of arithmetic tasks; only one patient achieved this. Figure 3a shows representative examples.

### Stratification by Injury Type

When stratified by injury type, differences persisted between patients with anoxic brain damage with both moderate and complex mental arithmetic. In patients with nonanoxic brain injuries, the differences were numerically, but not statistically, different (Table S5).

### Prolonged CMD

**Fig. 4:**
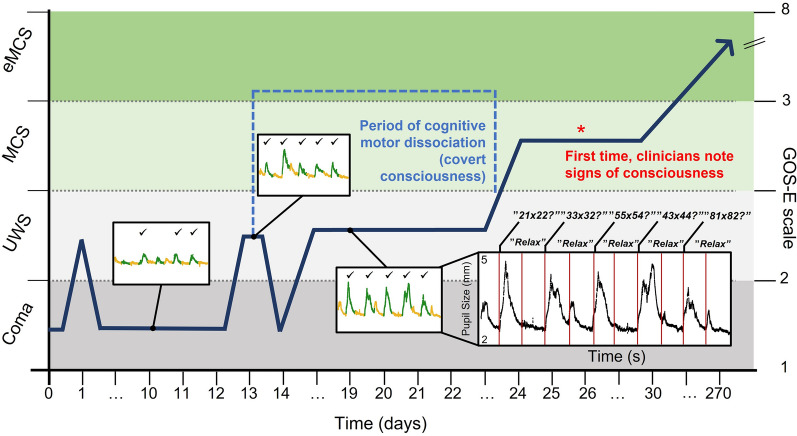
Covert consciousness (CMD) detected by automated pupillometry 2 weeks prior to overt command following. A 37-year-old previously healthy woman with subarachnoid hemorrhage was treated with clipping of the responsible pericallosal artery aneurysm. The diagram depicts the trajectory of consciousness recovery (dark blue arrow), including mental arithmetic-task-based pupillometry on days 10, 13, and 19. Between days 0 and 24, the patient’s clinical arousal level (*y*-axis) indicated she was in a coma or UWS. On day 10, mental arithmetic performance (3 of 5 pupillary dilations) reached the prespecified threshold for command following, indicative of CMD. The patient successfully followed commands also on days 13 and 19, as shown by 5/5 pupillary dilations. This period of covert consciousness lasted for almost 2 weeks (light blue dashed line). On day 26, the treating clinicians first noticed visual tracking and attempts to follow commands (red asterisk). At the 9-month follow-up, the patient could attend most activities of daily living (Glasgow Outcome Scale-Extended score of 5). Inserts: green lines of the plots represents the stimulation periods, whereas the yellow lines represent the rest periods in between tasks; check marks in the plots indicate the presence of pupillary dilations during mental arithmetic tasks; the *y*-axis depicts pupillary diameter in millimeters, and the *x*-axis is time in seconds. See also Fig. 3b. CMD, cognitive motor dissociation, eMCS, emerged from minimally conscious state, GOS-E, Glasgow Outcome Scale-Extended, MCS, minimally conscious state, UWS, unresponsive wakefulness syndrome (Color figure online)

In one behaviorally UWS patient with subarachnoid hemorrhage, repeated pupillary dilations during mental arithmetic were detected 2 weeks before overt command following noted by the attending clinicians, suggesting prolonged CMD (Figs. 3b and 4).

## Discussion

This is, to our knowledge, the first study investigating automated pupillometry combined with passive and active cognitive paradigms to discover residual consciousness in acute DoC. In contrast to functional magnetic resonance imaging (fMRI) and EEG-based consciousness paradigms, which are computationally complex, labor-intensive, and logistically demanding (precluding their routine use in the ICU [6]), pupillometry only requires a low-cost, handheld bedside device and a relatively simple data processing algorithm. Our key finding is that pupillometry together with mental arithmetic could detect covert consciousness in acute DoC, including persistent command-following behavior several days prior to overt awareness.

### Passive Cognitive Paradigms Cannot Distinguish Between Degrees of DoC

Although seeing one’s own facial reflection in a mirror caused pupillary dilation more than twice as often in conscious volunteers than in patients, this paradigm could not distinguish between patient groups. The auditory paradigm worked even worse; there were no differences between controls and patients. The most reasonable explanation is that pupillary dilation requires sustained arousal or prolonged mental activity that exceeds that provoked by passive visual and auditory stimuli. Although this may appear disappointing at first sight, there is also a very reassuring aspect to this negative finding, as explained in the next section on active paradigms.

### Active Cognitive Paradigms Can Differentiate Between Degrees of DoC

Pupillometry combined with mental arithmetic revealed strong differences in the response to cognitive loads, from absence of responses in most comatose patients and patients with UWS to presence of responses in most conscious volunteers, with patients with MCS ranging in between. Results remained consistent across sensitivity analyses using different thresholds for success. Reassuringly, given that passive paradigms did not result in meaningful group differences, we can confidently rule out that the pupillary responses seen with mental arithmetic were induced by passively hearing the instructions. Instead, they do seem to reflect true cognitive load and hence mental activity. Remarkably, in at least one behaviorally UWS patient, such mental activity was observed on three occasions almost 2 weeks before clinical signs of consciousness were noted by the attending clinicians, demonstrating the proof-of-concept that automated pupillometry paradigm can detect states suggestive of CMD.

The success rates of 62–88% in healthy volunteers are identical to those seen in active cognitive paradigms using fMRI [21] and EEG [22]. The low success rates contribute to decreased sensitivity, a limitation that also applies to fMRI-based and EEG-based paradigms. Not all healthy volunteers can maintain concentration long enough to participate successfully in these paradigms. The optimal threshold for success in terms of sensitivity and specificity remain unknown. There is no gold standard to measure consciousness [23], but CMD occurs in 15–20% of patients with acute [3, 8] and chronic [2] DoC, suggesting that ≥ 4 dilations in the moderate or the hard mental arithmetic task may be the best threshold.

Although our method is unlikely to have identified every patient with residual or covert consciousness, we think it potentially provides clinically meaningful positive predictive values and specificities. A caveat is that the positive results mainly came from patients with anoxic brain injury. Whether pupillometry with mental arithmetic works equally well in acute DoC caused by traumatic and other nonanoxic brain injuries remains to be seen. Notably, however, pupillometry with mental arithmetic appears to have greater potential than pupillometry combined with the auditory “local global” paradigm [24], probably because of the larger cognitive load that comes with mental arithmetic. Head-to-head studies comparing automated pupillometry with fMRI-based and EEG-based active paradigms are required to investigate this further. In the meantime, we provide detailed advice on how to replicate our methodology, including a preregistered statistical analysis plan, video instructions regarding the examination technique, and open access to the code and algorithm required to process the pupillometry data.

## Conclusions

Automated pupillometry combined with mental arithmetic can discover residual consciousness in patients with acute DoC, potentially including those with CMD. Given that fMRI-based and EEG-based paradigms to identify covert consciousness depend on sophisticated computational expertise and are logistically challenging in the ICU, we think the convenience of this low-cost bedside paradigm makes it a promising novel biomarker in acute DoC.

### Supplementary Information

Below is the link to the electronic supplementary material.

Supplementary file1 (DOCX 790 KB)
